# Genetic factors in rotator cuff pathology: potential influence of col 5A1 polymorphism in outcomes of rotator cuff repair

**DOI:** 10.1186/s12881-020-01022-0

**Published:** 2020-04-17

**Authors:** Stefano Petrillo, Umile Giuseppe Longo, Katia Margiotti, Vincenzo Candela, Caterina Fusilli, Giacomo Rizzello, Alessandro De Luca, Vincenzo Denaro

**Affiliations:** 1 Prosthetic Surgery Centre, IRCCS Orthopedic Institute Galeazzi, via Riccardo Galeazzi 4, 20161 Milan, Italy; 2 Department of Orthopaedics and Trauma Surgery, Campus Bio-Medico University of Rome, Rome, Italy; 3 Laboratory of Molecular Medicine and Biotechnology, Campus-Bio Medico University of Rome, Rome, Italy; 4 Molecular Genetics Unit, Casa Sollievo della Sofferenza Hospital, IRCCS, San Giovanni Rotondo, Italy; 5 Istituto Casa Sollievo della Sofferenza - Mendel, Viale Regina Margherita 261, 00198 Roma, Italy; 6 ALTAMEDICA, Laboratorio Genetica Medica, Roma, Italy; 7 Bioinformatics Unit, Casa Sollievo Della Sofferenza Hospital, IRCCS, San Giovanni Rotondo, Italy; 8 UOS Diagnosi Genetica Molecolare Istituto CSS-Mendel, Viale Regina Margherita 261, 00198 Roma, Italy

**Keywords:** Rotator cuff, genetic, COL5A1 polymorphism, Outcomes, Shoulder, Arthroscopy

## Abstract

**Background:**

Investigations in genetics have provided valuable information about the correlation between gene variants and tendinopathy. Single Nucleotide Polymorphisms of COL5A1 gene are reported to be involved in Achilles tendinopathy, chronic degenerative tendon changes at the elbow, and other tendinopathies. The influence of Single Nucleotide Polymorphisms of COL5A1 was previously analyzed in rotator cuff disease with confounding results. Moreover, the rs12722 polymorphism in COL5A1 gene has been implicated in the aetiology of musculoskeletal soft tissue injuries in several association studies. This study aims to analyse the possible influence of rs12722 polymorphism in COL5A1 in the outcomes of rotator cuff repair.

**Methods:**

Seventy-nine patients were included in the study. DNA was extracted from 1.2 ml of venous blood and genotyped for COL5A1 SNPs rs12722. Rotator cuff muscle strength and range of motion (ROM) in anterior elevation, external and internal rotation of the shoulder were evaluated.

**Results:**

Patients presenting COL5A1 SNP rs12722 CC showed a ROM of passive external rotation statistically significantly higher compared to patients with CT genotype and TT genotype.

**Conclusions:**

COL5A1 SNP rs12722 may influence the functional outcomes of RCRs, even though further studies are required to confirm these preliminary results.

## Background

Rotator cuff (RC) tears are common diseases that affect especially the middle age individuals [1–6]. Approximately 30 to 50% of people older than 50 years are affected by a RC disorder, and these percentages increase with aging [7, 8].

Despite several valuable therapeutic and surgical strategies were developed in the management of RC pathologies [9–12], their etiology and pathogenesis are still unclear [13–19]. Intrinsic, extrinsic, and biological factors contribute to RC tears [1]. Moreover, when considering the outcomes of RC repair (RCR) procedures, surgical and nonsurgical factors [20–23] may affect tendon healing after the surgical repair.

In the last decade, several authors have shifted their attention to the identification of genetic factors involved in RC disorders [24, 25]. Peach et al. [26] reported a significant correlation between RC tears and variants of different genes (ANHK and TNAP) involved in the metabolism of extracellular matrix (ECM) of the tendons. Supporting the role of ECM, fifteen single nucleotide polymorphisms (SNPs) were identified in the Tenascin-C (TNC) gene [27], which encodes for TNC protein, in patients with RC tears. This protein has a critical role in ECM homeostasis. It is over-expressed in injured tissues. Moreover, other authors analysed the genes encoding for estrogenic-related receptor-beta (ESRRB). ESRRB is a nuclear receptor that has a role in promoting cell survival in hypoxic environments. SNP variant in the ESRRB gene was observed in patients who had lateral failure after rotator cuff repair compared with those that healed [28, 29].

Motta et al. [30] demonstrated that variants of SNPs in genes encoding for fibroblastic growth factors (FGFs) and defensin-beta 1 (DEFB1), which are both immune-modulating proteins, are significantly associated with RC tears.

Moreover, the role of variants of collagen type V alpha 1 (COL5A1) gene in RC tears was already evaluated in the onset of RC pathology [27]. COL5A1 gene encodes for the alpha one chain of type V collagen (OMIM 120215), which is a key protein in the regulation of type I collagen fibrils diameter and also a major constituent of tendons and ligaments bundles [31]. It has been shown that Achilles and elbow tendinopathies were associated with different SNPs of COL5A1 gene [32, 33].

No studies analyzing the potential influence of SNPs of COL5A1 in the outcomes of RCR procedures are available in the current literature. The present study aims to determine whether a DNA variant of COL5A1 rs12722 CT can influence the clinical and functional outcomes after RCR procedures.

## Methods

The present study was approved by the local Ethics committee of Campus Bio-Medico of Rome. All participants were recruited at the University Hospital Campus Bio-Medico of Rome. Written informed consent was obtained from all participants.

### Patient recruitment

Two hundred patients who consecutively underwent arthroscopic rotator cuff repair at our institution between January 2011 and December 2012 were evaluated. Patients with massive RC tears according to Cofield classification were excluded from this study to avoid any potential bias. Other preoperative/intraoperative and postoperative inclusion and exclusion criteria of the study are summarized in Table 1. Nineteen patients were excluded for associated gleno-humeral instability with capsule-labral pathologies; 2 patients were excluded for fracture of the glenoid or the greater/lesser tuberosity of the humerus; 5 patients were excluded for inflammatory joint disease; 17 patients were excluded for gleno-humeral osteoarthritis; 14 patients were excluded for RC arthropathy; 49 patients were excluded for irreparable massive RC tears; 15 patients were excluded for bilateral RC disease. A total of 79 patients with RC tears were enrolled. The average follow-up was 47.9 + 24.1 months (range 14–124; median 52 months).

**Table 1:** Inclusion and Exclusion criteria

	Patients enrolment	
	Preoperative/Intraoperative	Postoperative
**Inclusion criteria**	Diagnosis of RC tear: clinical grounds, MRI evidence of full-thickness RC tear, Symptoms: history of shoulder symptoms refractory to non-operative management (non-steroidal anti-inflammatory drugs or injections, physiotherapy, rest)	Arthroscopic bilateral RC repair procedures; minimum follow-up of 1 month after surgery
**Exclusion criteria**	Associated pathologies of the shoulder: gleno-humeral instability with capsule-labral pathologies; fracture of the glenoid or the greater/lesser tuberosity of the humerus; inflammatory joint disease, glenohumeral osteoarthritis, RC arthropathy. Irreparable RC tears. Bilateral RC disease	Follow-up < 12 months; inability to complete clinical questionnaires due to language problems or cognitive disorders

DNA was extracted from a 1.2 ml of venous blood. Patients were allocated to each study group according to results of rs12722 SNP genotype of COL5A1: Group 1 (CC), Group 2 (CT); Group 3 (TT).

### DNA extraction and genotyping

DNA was extracted from approximately 1.2 ml of venous blood through the MagCore extractor system H16 with a MagCore Genomic DNA Large Volume Whole Blood Kit (RBC Bioscience Corp, Taipei, Taiwan). SNPs rs12722 was genotyped through ready-to-use TaqMan assay (Life Technologies, Carlsbad, California, USA) on an HT 7900 platform (Life Technologies, Carlsbad, California, USA). Primer Express synthetized PCR primers and TaqMan probes optimizing them in accordance to the manufacturer’s protocol. The Genotyping quality test was performed through 12 blinded duplicate samples (four duplicates for each rs12722 SNP genotype: CC, CT, TT) in each 384-well assay. The average percentage of agreement of duplicate samples was > 99.

### Clinical and functional assessment

The clinical and functional outcomes were assessed by two blinded examiners at the last follow-up evaluation and included modified University of California, Los Angeles (UCLA) shoulder score [34], Oxford shoulder score (OSS) [35, 36], measurement of passive-active range of motion (ROM) and muscle strength of the operated shoulder. Inter-observer agreement was assessed (kappa coefficient for inter-observer agreement 0.82). According to standard measurement guidelines [37], passive and active anterior elevation (sagittal plane), internal rotation and external rotation (90° abduction) were measured by a universal goniometer with the patients in supine position.

RC muscles strength in anterior elevation, external and internal rotation of the shoulder was evaluated using a CH15K20-KERN dynamometer (KERN & Sohn GmbH, Balingen, Germany), and the results were expressed in newton (N).

Both examiners repeated three times the measurements of each ROM and strength. Statistical analysis was performed on the average value for each variable.

Inter-observer agreement was performed (kappa coefficient 0.82).

### Arthroscopic technique

The same surgeon performed all the surgical procedures with a standardized surgical technique. The patient was positioned in beach chair under combined brachial plexus block and general anesthesia. Five kilograms of traction were used to obtain shoulder distraction. The long head of the biceps was tenotomised in all patients. Then, through the lateral portal, the RC was mobilized to its bony insertion, and RC footprint was abraded with a burr. RCR was performed through one row of suture anchors double-loaded.

### Postoperative management

The same supervised rehabilitation protocol for 6 months after surgery was used for all patients. A sling with an abduction pillow was used for 6 weeks. Passive external rotation was started from the first day after surgery. Overhead stretching was not allowed until 6 weeks after surgery to prevent damage to the repair. The sling was removed in the sixth week. At 10–12 weeks after the surgical procedure, physical sessions including rehabilitation of the RC, deltoid and scapular stabilizers and, isoinertial strengthening were initiated. Rehabilitation lasted 6 months. Overhead activities or heavy manual work were allowed 6 months after surgery.

### Statistics

The 19.0 version of SPSS (Chicago, Illinois) was used to perform all the statistical evaluations considering the total modified UCLA shoulder score, and total OSS. We also considered passive and active ROM of the shoulder as well as strength of anterior elevation, internal rotation, and external rotation ROM of the shoulder.

The outcome variables (i.e., shoulder outcome scores, active and passive ROM, muscle strength) were compared using ANOVA; the difference was considered statistically significant for *p*-values < 0.05.

A post hoc power analysis using ER ROM was performed. The post hoc power analysis, based on allele frequency (National Center for Biotechnology Information. ClinVar; [VCV000365746.1], https://www.ncbi.nlm.nih.gov/clinvar/variation/VCV000365746.1), showed that the power of the study is 90% with an effect size of 0.458.

## Results

### Complications

No patients developed complications after surgery, as well as clinical symptoms of RC re-tears at the last follow-up.

### Demographics

Overall, the study included 79 patients (42 females; 37 male) with a mean age of 61 + 9.8 years (range 31–79; median 63 years) (Table 2). The population tested is in Hardy-Weinberg equilibrium (*p* = 0.908775).

**Table 2:** Demographic characteristics of the patients

Demographics	Group 1 (12 pz)	Group 2 (39 pz)	Group 3 (28 pz)
**Male:Female**	5:7	17:22	15:13
**Mean Age + standard deviation**	59.2 + 10.6	62.3 + 10.1	59.9 + 9.2
**Dominant arm involved (%)**	8 (61.5%)	26 (70%)	20 (71.4%)
**Mean follow up (months)** **+** **standard deviation**	52.4 + 7.8	47.1 + 25.3	47.3 + 25.1

Legend: Pz, Patients

### Clinical and functional outcomes

The mean modified UCLA score was 29.1 + 6.4 points (range 16–35; median 30.5 points) in group 1, 26.9 + 7.2 points (range 6–35; median 28 points) in group 2 and 25.1 + 7.5 points (range 10–35; median 28 points) in group 3. The mean OSS was 19.6 + 8.1 points (range 12–35; median 18 points) in group 1, 23.2 + 12.1 points (range 12–53; median 18 points) in group 2 and 24.3 + 9.9 points (range 12–51; median 21.5 points) in group 3.

According to modified UCLA shoulder score, results of RCR were excellent in 3 (25%) patients of group 1, 5 (12.8%) patients of group 2 and 3 (10.7%) patients of group 3; good in 6 (50%) patients of group 1, 12 (30.8%) patients of group 2 and 12 (42.8%) patients of group 3; fair in 1 (8.3%) patients of group 1, 12 (30.8%) patients of group 2 and 5 (17.8%) patients of group 3; poor in 2 (16.7%) patients of group 1, 6 (15.6%) patients of group 2 and 8 (28.7%) patients of group 3.

The comparison among clinical and functional outcomes results through the three groups of patients was showed in Table 3.

**Table 3:** Statistical comparison of clinical outcomes between the 3 groups

Scores	Result of ANOVA (***p***-value)
UCLA	0,26
OSS	0,45
A.E. PASSIVE	0,45
E.R. PASSIVE	0,05*
I.R. PASSIVE	0,07
A.E. ACTIVE	0,4
E.R. ACTIVE	0,12
A.E. STRENGHT	0,23
E.R. STRENGHT	0,3
I.R. STRENGHT	0,7

Legend: *UCLA* University of California, Los Angeles modified shoulder score, *OSS* Oxford shoulder score. *AE* Anterior elevation, *ER* External rotation, *IR* Internal rotation. *: statistically; *ANOVA* Analysis of variance

Table 4 reports the mean values of passive and active ROM and strength of the operated shoulder in each group of patients.

**Table 4:** Functional outcomes

	Passive range of motion ^**o**^			Active range of motion ^**o**^		Strength (N)		
	***AE***	***ER***	***IR***	***AE***	***ER***	***AE***	***ER***	***IR***
**Group 1**	174.2 + 14.4	78.3 + 18.9*	89.2 + 2.9	171.6 + 15.9	52.9 + 17.6	52.9 + 25.5	52.9 + 24.5	70.5 + 25.5
**Group 2**	173.1 + 21.3	73.5 + 23.1*	82.8 + 4.8	161.1 + 30.7	44.5 + 18.3	41.2 + 20.6	45.1 + 19.6	63.7 + 25.5
**Group 3**	167.1 + 22.4	60.7 + 27.2*	75.2 + 25.3	158.6 + 28.9	40.2 + 16.9	42.1 + 22.5	42.1 + 16.7	63.7 + 27.4

Legend: Results reported as mean value + standard deviation; ^o^: degrees; *N* Newton, *AE* Anterior elevation, *ER* External rotation, *IR* Internal rotation. *: statistically significant

## Discussion

This was the first study to have analysed the potential influence of SNPs of COL5A1 in the outcomes of RC repairs. The present results suggested that COL5A1 SNPs rs12722 might influence the functional outcomes of RCR procedures. Patients presenting COL5A1 SNP rs12722 CC (group 1) showed a ROM of passive external rotation statistically significantly higher compared to patients with CT genotype (group 2) and TT genotype (group 3).

When considering clinical outcomes after RCR, according to modified UCLA shoulder score, good to excellent results were found in 75, 43.6 and 53.5% of patients of group 1, 2 and 3, respectively. However, these differences were not statistically significant. Furthermore, no significant differences in OSS and muscle strength results were found between the three groups of patients.

The interest in COL5A1 gene raises from previous studies reporting on a correlation between different SNPs of such gene and tendinopathies. Mokone et al. [32] was the first detecting an influence of SNPs of COL5A1 gene in Achilles tendinopathy in a cohort of 240 patients, reporting significant differences in the allele frequencies of the COL5A1 between the two groups. Similar results were found by Altinisik et al. [33] in patients with chronic degenerative tendon changes at the elbow. However, these findings were not confirmed by Kluger et al. [27] in two different cohorts of patients with RC tears (1st: 59 patients with RC tears and 32 controls; 2nd: 96 patients with RC tears and 44 controls).

Several factors are involved in the aetiology and pathogenesis of RC disorders. An increased prevalence rate of RC tears was demonstrated in females, in Caucasians and individuals older than 50 years [1]. Overhead sports athletes such as baseball, volleyball and tennis athletes or hard manual workers have a high incidence of RC tears or tendinopathy, even at a young age [18]. The prevalence of rotator cuff tendinitis ranged from 1% among data entry operators to 69% among industrial workers working above shoulder height [38] Moreover, obesity and metabolic dysfunctions were involved in RC tears [16, 39]. After rotator cuff repair, a BMI higher than 30 causes less favourable results in the Constant and DASH scores and showed higher re-tear rates [40]. Moreover, advanced glycation end products are supposed to be involved in age-related degenerative rotator cuff changes [41].

Recently, investigations in genetics have provided valuable information about the correlation between gene variants and RC tears. Two different genes, *ANHK* and *TNAP,* which are involved in the metabolism of ECM of the tendons, variants of genes encoding for estrogenic-related receptor-beta (ESRRB), responsible during tissue hypoxia, were related to RC tears [26, 29]. Variants in ANKH and TNAP alter extracellular inorganic pyrophosphate concentrations and cause calcium crystal deposition, leading to rotator cuff arthropathy [26]. Moreover, two SNPs, residing in SAP30BP on chromosome 17 and SASH1 on chromosome 6, implicated in the cellular process of apoptosis, were significantly associated with rotator cuff tears [29]. Furthermore, 15 SNPs were identified in patients with RC tears in the Tenascin-C (TNC) gene [27]. This gene encodes for TNC protein, which is crucial in ECM synthesis and organization after tendon tissue damages. Motta et al. [30] and Teerlink et al. [42] have been demonstrated the role of ESRRB in RC tears, also showing that genetic variants of *ESRRB* gene were associated with failure of RC healing.

Surgical and nonsurgical factors influence the results of RCR repair procedures, as well as capacities of the torn tendons to heal. Patients with large or massive tears, chronic lesions, muscle atrophy or fatty degeneration of the RC muscles have worst outcomes [43]. Moreover, 16% of failed RCR were related to postoperative rehabilitation [44]. Cummins et al. [45] assessed that suture pulling through the repaired tendon was the most common cause of failure during the revision of RC repair. On the other hand, Park et al. [46] highlighted the external rotation after RCR as responsible for producing a gap formation in the anterior portion of the supraspinatus tendon. At the same time, genetics, and in particular family history of RC tear increases the risk of healing failure [28, 29]. Probably, following the results of our investigation and the study of Teerlink et al. [42], also genetics factors should be taken into account when considering factors affecting outcomes of RCR procedures.

The C-to-T rs12722 polymorphism is reported to be associated with altered stability of COL5A1 mRNA, encoding for alpha 1 chain of collagen type V, a quantitatively minor fraction fibrillar collagen involved in the regulation of collagen fibril assembly [47]. The COL5A1 3′-UTR with the rs12722 polymorphism T allele exhibited enhanced mRNA stability compared to the 3′-UTR of the C allele. This data suggest that T allele is involved in the synthesis of more type V collagen α1 chain than other polymorphism, resulting in changes in the mechanical properties of the connective tissue [48]. In this study patients with TT genotype showed a ROM of passive external rotation statistically significantly lower compared to other analyzed genotypes, supporting the hypothesis that the T allele of the *COL5A1* rs12722 polymorphism is associated with high muscle stiffness [49]. ClinVar database characterized COL5A1 rs12722 polymorphism as benign (National Center for Biotechnology Information. ClinVar; [VCV000365746.1], https://www.ncbi.nlm.nih.gov/clinvar/variation/VCV000365746.1)​.

The main two strengths of our study are that we have enrolled a homogeneous sample (only Caucasian) and that all the genetics evaluations were performed by an expert geneticist, who has relevant experience in the field of genetic analysis.

Other strengths are: 1) all the surgical procedures were performed by a single shoulder surgeon using a well-defined technique; 2) no patients experienced surgical complications; 3) the follow up evaluations were performed blindly by two independent examiners; 4) the length of follow up is adequate to consider the results of surgery as well-established 5) the evaluation of ROM and strength was performed according to standard measurement guidelines; 6) patients with massive RC tears and bilateral RC pathology were excluded; 7) a professional physiotherapist followed all the patients during the entire rehabilitation period.

Nevertheless, the most significant limitation of our study is that we have tested only the COL5A1 SNP rs12722. Therefore, we are aware that other genetic variations may be potentially related to RC tears. Other limitations are the absence of diagnostic images of the operated shoulder at the time of functional assessment and the absence of functional data from the unaffected shoulder for comparison with the operated side. Further studies are required to evaluate the unaffected shoulder considering the possible effects of whether the dominant vs non-dominant shoulder was affected.

This study should be considered as preliminary evidence of a potential role of Col5A1 SNPs rs12722 in determining the outcome of RC procedures.

## Conclusions

Col5A1 SNPs rs12722 may influence the functional outcomes of RCR procedures, even though further studies are required to confirm these preliminary results.

## Data Availability

All data generated or analysed during this study are included in this published article.

## Abbreviations

RC: Rotator cuff
RCR: RC repair
ECM: Extracellular matrix
SNPs: Single nucleotide peptide polymorphisms
TNC: Tenascin-C
ESRRB: Estrogenic-related receptor-beta
DEFB1: Defensin-beta 1
COL5A1: Collagen type V alpha 1
UCLA: University of California, Los Angeles
OSS: Oxford shoulder score
ROM: Range of motion
N: Newton
RFLPs: Restriction fragment length polymorphisms
DASH: Disabilities of the Arm, Shoulder and Hand
